# Active Learning‐Assisted Exploration of [PO_40_Mo_12_]^3−^ for Alzheimer's Therapy Insights

**DOI:** 10.1002/advs.202508702

**Published:** 2025-08-19

**Authors:** Lincan Fang, Ruoxue Peng, Luping Xia, Gui‐lin Zhuang

**Affiliations:** ^1^ Key Laboratory of Functional Molecular Solids Ministry of Education College of Chemistry and Materials Science Anhui Normal University Wuhu Anhui 241002 P.R. China

**Keywords:** active learning, alzheimer, bayesian optimization, DFT, POM

## Abstract

Alzheimer's disease (AD), involving amyloid‐β (Aβ) aggregation, has potential therapeutic modulators in polyoxometalates (POMs) like [PMo_12_O_40_]^3−^. To clarify their inhibitory mechanisms, a multiscale computational strategy integrating active‐learning Bayesian Optimization (BO) and density functional theory (DFT) is employed to explore low‐energy configurations of isolated amino acids, [PMo_12_O_40_]^3^^−^–amino acid complexes, and [PMo_12_O_40_]^3^^−^–peptide systems. Hydrogen bonding and Coulombic repulsion dominate adsorption stability. Crucially, oxygen atoms in the [PMo_12_O_40_]^3^^−^ cluster form multiple weak interactions (e.g., van der Waals, hydrophobic) with alkyl side‐chain hydrogens in Aβ peptides. The synergistic effect of these weak interactions induces robust binding between the POM and peptide chains, stabilizing a tightly bound complex that sterically hinders Aβ self‐assembly. Notably, simulations predict that the cluster preferentially targets hydrophobic amino acids with alkyl chains (valine, lysine, leucine, isoleucine) located in Aβ regions critical for aggregation—specifically, namely Aβ12, Aβ16‐18, Aβ24, Aβ28, Aβ31‐32, Aβ34‐36, and Aβ39‐41. These insights highlight the role of multivalent weak interactions in POM‐mediated inhibition and identify key interfacial residues for therapeutic targeting.

## Graphical abstract

Bayesian Optimization coupled with active learning to uncover the interaction mechanism of [PO_40_Mo_12_]^3−^ on amyloid‐β peptide.

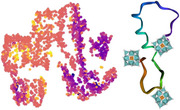

## 1 Introduction

As one of modern medicine's most formidable and enigmatic challenges, Alzheimer's disease (AD) is a progressive neurodegenerative disorder characterized by memory loss and cognitive decline, significantly impacting the quality of life for millions of elderly individuals worldwide.^[^ ^1^ ^]^ Despite many controversies, a central event in the pathogenesis of AD is the aggregation of amyloid‐β (Aβ) peptides into amyloid fibrils, a process that has been extensively studied.^[^ ^2^, ^3^, ^4^ ^]^ In recent developments, the U.S. Food and Drug Administration approved three anti‐amyloid monoclonal antibodies—aducanumab, lecanemab, and donanemab—for the treatment of early‐stage AD.^[^ ^5^ ^]^ These antibodies have demonstrated the ability to clear Aβ plaques and slow cognitive decline in patients with early‐stage AD, thus supporting the therapeutic potential of targeting Aβ.^[^ ^6^ ^]^ As a result, regulating the aggregation of Aβ is now considered a promising approach in the ongoing battle against AD.

The latest years have witnessed that the exploration of metal‐based compounds, particularly POMs, as metallodrug agents for treating various diseases has garnered increasing attention.^[^ ^7^, ^8^, ^9^ ^]^ Essentially, Polyoxometalates (POMs) are promising modulators of amyloid‐β (Aβ) aggregation, leveraging tunable physicochemical properties (such as redox potentials, acidity, polarity, and surface charge) to therapeutically target Aβ‐related pathologies. In this vein, Liu et al. demonstrated that the spherical morphology of POMs exhibited significant inhibitory effects on Aβ aggregation.^[^ ^10^ ^]^ Notably, research by Qu et al. thoroughly revealed that the wheel‐shaped structure POM, P_5_W_30_, interacted with Aβ peptides, effectively preventing their fibrillization.^[^ ^11^ ^]^ Additionally, Hureau's group found that lacunary POMs with a Keggin structure were capable of removing Cu^2+^ ions bound to Aβ, halting the Aβ–Cu^2+^ complex‐induced generation of reactive oxygen species, and thereby modulating Aβ aggregation.^[^ ^12^ ^]^ These findings underscore the therapeutic potential of POMs in the context of Alzheimer's disease. Despite their therapeutic potential for AD, POMs face drug development challenges due to poorly understood atomic‐level interactions with proteins, peptides, and amino acids, which obscure their mode of action and hinder rational design.^[^ ^13^ ^]^

**Figure 1:**
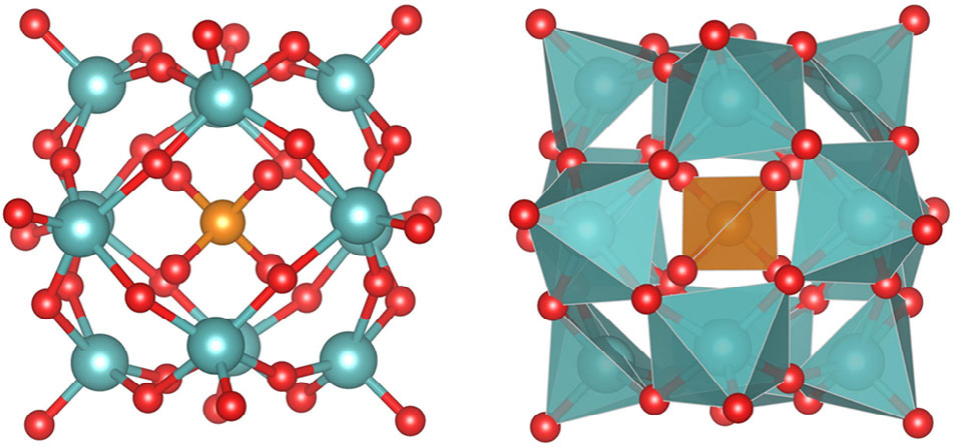
The ball‐stick model (left) and polyhedral model (right) of [PO_40_Mo_12_]^3−^. Red color is used for oxygen atoms, dark orange for phosphorus, and cyan blue for molybdenum.

Experimentally, cryo‐electron microscopy (Cryo‐EM) revolutionizes the study of atomic‐level interactions between POMs and Aβ fibrils, yet its widespread application is hindered by experimental challenges: sample heterogeneity, low contrast, beam sensitivity, dynamic interactions, artifact risks, and data interpretation complexity.^[^ ^14^, ^15^ ^]^ Fortunately, computational simulations, particularly the first‐principles DFT method, provide atomic‐level insights into the microscopic structure of adsorbate systems, offering resolution beyond the capabilities of experimental techniques.^[^ ^16^, ^17^, ^18^, ^19^ ^]^ For large molecules (e.g., POMs, biomacromolecules), sole DFT‐based determination of adsorption mechanisms is challenged by complex phase spaces and high computational costs. Recently, various approaches have been proposed to tackle these challenges, including systematic, stochastic, hierarchical, and machine‐learning techniques.^[^ ^20^, ^21^, ^22^, ^23^, ^24^, ^25^ ^]^ Within this context, Bayesian Optimization (BO), as a probabilistic global optimization method, has emerged as a powerful and low‐cost tool for structure search in an interaction system when combined with DFT, enabling efficient exploration of the complex potential energy surface (PES).^[^ ^26^ ^]^ Previous studies demonstrated that the Bayesian Optimization Structure Search (BOSS) code,^[^ ^27^ ^]^ integrated with DFT, efficiently identifies stable structures of amino acids and ligands bound to gold nanoclusters.^[^ ^28^, ^29^ ^]^ The BOSS framework iteratively constructs a surrogate PES via BO sampling. Upon convergence, local minima from the surrogate PES are refined with DFT, validating its accuracy in exploring molecular and adsorbate systems.

Although BOSS has proven effective in small‐molecule and nanocluster systems, its application to biologically complex, hybrid bio‐inorganic interfaces remains unexplored. Addressing this gap, we herein applied BOSS for the first time, in combination with an active‐learning strategy, to investigate the interaction between the highly charged POM cluster ([PO^4^₀Mo₁^2^]³^−^, as shown in Figure **1**1) and intrinsically disordered Aβ‐related peptides, a system of considerable therapeutic relevance. This hybrid interface, featuring a large, multi‐valent inorganic cluster and a flexible biomolecular chain, poses significant challenges for structural sampling and energetic evaluation. The bottom‐up simulations uncovered low‐energy structures of key (Aβ‐related) amino acids and peptide chains, dynamic adsorption configurations, and the specific interaction mechanisms within the POM–amino‐acid and POM–peptide‐chain complexes. The results reveal preferential binding modes between POM and specific hydrophobic residues in Aβ peptides, demonstrating how synergistic weak interactions create strong POM‐peptide binding.Critically, this stabilizes a complex that sterically blocks Aβ self‐assembly, shedding light on how these cooperative effects can potentially hinder fibril formation. This application not only demonstrates the robustness of BOSS in addressing high‐dimensional biological systems but also provides valuable atomistic insight into the inhibition mechanism of amyloid aggregation, offering theoretical guidance for the rational design of POM‐based therapeutics against Alzheimer's disease.

## 2 Results and Discussion

### 2.1 Low‐Energy Structures of Four Amino Acids Related to Aβ Peptides

**Figure 2:**
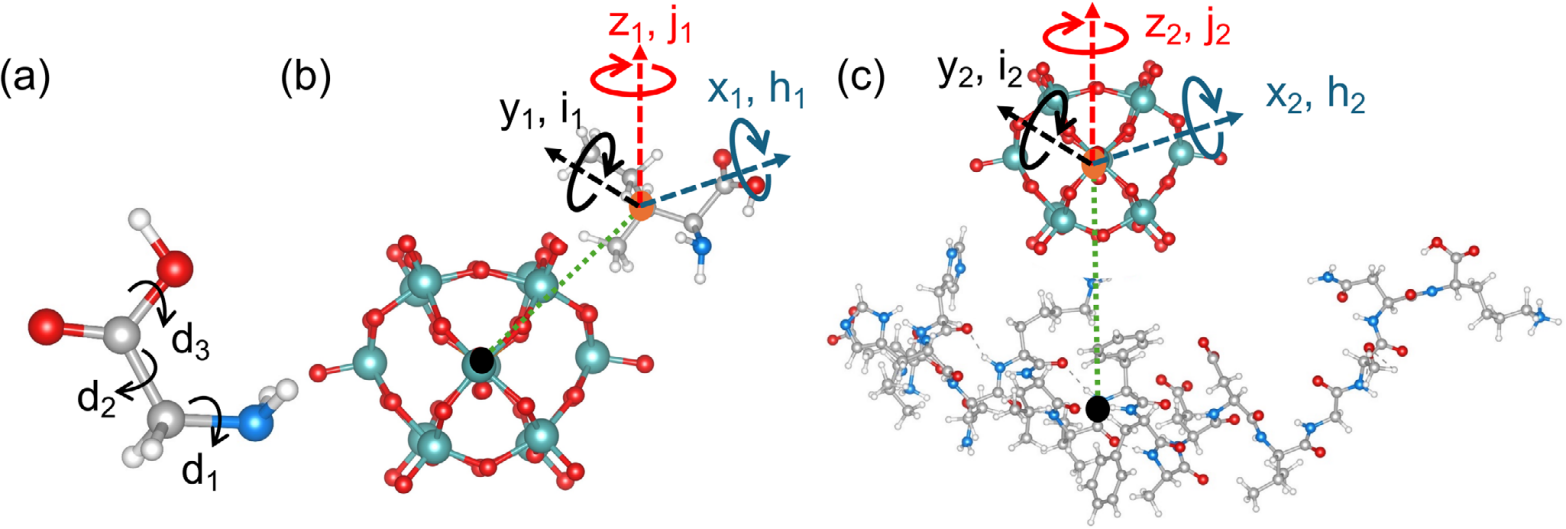
a) Ball‐stick model of an isolated Gly molecule. Red color is used for oxygen atoms, gray for carbon, white for hydrogen, and blue for nitrogen. d_1_, d_2_, and d_3_ label the three dihedral angles of glycine that we used to define our search space. b) Ball‐stick model of P‐Iso. x_1_, y_1_, z_1_, h_1_, i_1_, and j_1_ label the search space. c) Ball‐stick model of a [PO_40_Mo_12_]^3−^‐peptide‐chain interaction system. x_2_, y_2_, z_2_, h_2_, i_2_, and j_2_ label the search space.

To construct low‐energy adsorbate systems of [PO_40_Mo_12_]^3^^−^ with four amino acids (isoleucine, glycine, leucine, and methionine), we integrated the BOSS strategy with DFT calculations. Initial molecular geometries of the isolated amino acids were retrieved from the DrugBank database and pre‐optimized via DFT. Key dihedral angles, defining rotational flexibility while preserving DFT‐relaxed bond lengths and bond angles, were selected to parameterize the search space for each molecule, resulting in dimensions of 6, 3, 5, and 6 for Iso, Gly, Leu, and Met, respectively (**Figure** **2**a; Figure S1, Supporting Information). BO was then employed to efficiently explore the PES of each system, utilizing Gaussian‐process‐based surrogate models and acquisition functions to converge toward low‐energy configurations. This approach prioritized sampling regions of high uncertainty and energetic promise, identifying critical local minima while minimizing costly DFT evaluations. Finally, first‐principles DFT re‐optimization was performed on the BO‐derived structures to refine atomic coordinates and eliminate duplicates, ensuring both computational efficiency and physical accuracy. This hybrid workflow demonstrates how BO‐guided dimensionality reduction and intelligent sampling can address the challenges of high‐dimensional conformational searches in complex molecular systems.

The 29 local minima for Iso, 10 for Gly, 33 for Leu, and 38 for Met were identified with relative energies confined within 0.37, 0.47, 0.41, and 0.45 eV from their respective global minima. Obviously, both Iso and Gly exhibit higher conformational stability, while Leu and Met display greater structural flexibility, as evidenced by their broader energy distribution. If using a cutoff distance of r_HB_ = 3 Å to define hydrogen bonding, intramolecular hydrogen bonds between the amine and carbonyl groups were observed in all local minimum structures in each amino acid system. These interactions, particularly the type II hydrogen bonds (N··· H─O), play a critical role in stabilizing the low‐energy structures of the amino acids.

**Figure 3:**
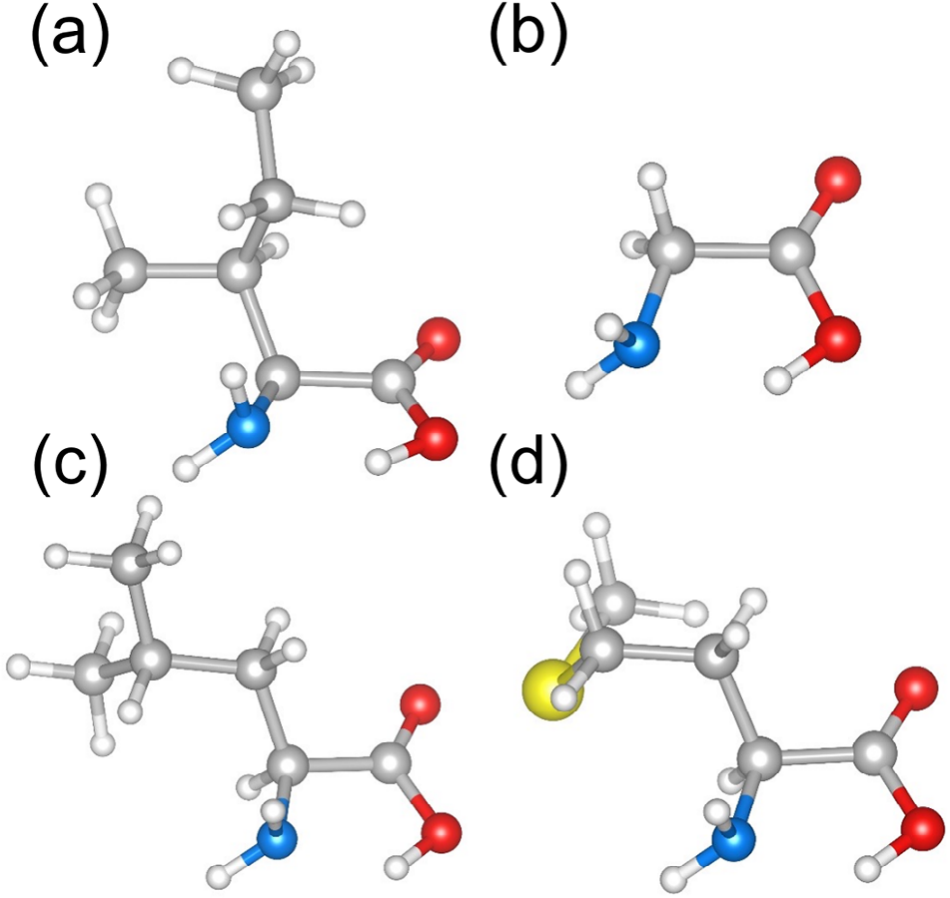
BOSS‐predicted lowest‐energy conformers of a) Iso, b) Gly, c) Leu, and d) Met.

As shown in Figure S2 (Supporting Information), the energy profiles of Iso and Gly reveal a high degree of conformational stability, with energy distributions primarily concentrated in lower energy ranges and exhibiting minimal fluctuations. Conversely, Leu and Met display greater variability in their energy levels, with broader distributions of conformational states, indicating higher structural flexibility and a propensity for conformational adaptation. These suggest that Iso and Gly are more likely to maintain fixed conformations under physiological conditions, whereas Leu and Met may exhibit a wider array of structural states, reflecting enhanced adaptability to varying environmental conditions. As depicted in **Figure** **3**, the predicted lowest‐energy structures of Iso, Gly, Leu, and Met feature an II hydrogen bond. The II hydrogen bond contributes significantly to the stabilization of these conformations, underscoring its critical role in the structural integrity of the amino acids. These four amino acid molecule structures were used for the next study.

### 2.2 [PO_40_Mo_12_]^3−^‐Amino‐Acid Interaction Systems

Using the lowest‐energy conformers of Iso, Gly, Leu, and Met, four adsorbate systems with [PO_40_Mo_12_]^3−^ named P‐Iso, P‐Gly, P‐Leu, and P‐Met, were constructed as shown in Figure 2b and Figure S3 (Supporting Information). In these systems, the [PO_40_Mo_12_]^3−^ was modeled based on a previous study, while the amino acid structures were derived from BOSS‐predicted lowest‐energy conformers. Therefore, when defining the search space for each system, the structure of [PO_40_Mo_12_]^3^^−^ and the amino acid molecule were kept fixed, while only the adsorbate position—specified by the coordinates of the center of mass of the amino acid molecule (x_1_, y_1_, z_1_)—and molecular rotational degrees of freedom (h_1_, i_1_, j_1_) were allowed to vary. The BOSS‐based procedure was utilized to actively learn the PES for each system.

**Figure 4:**
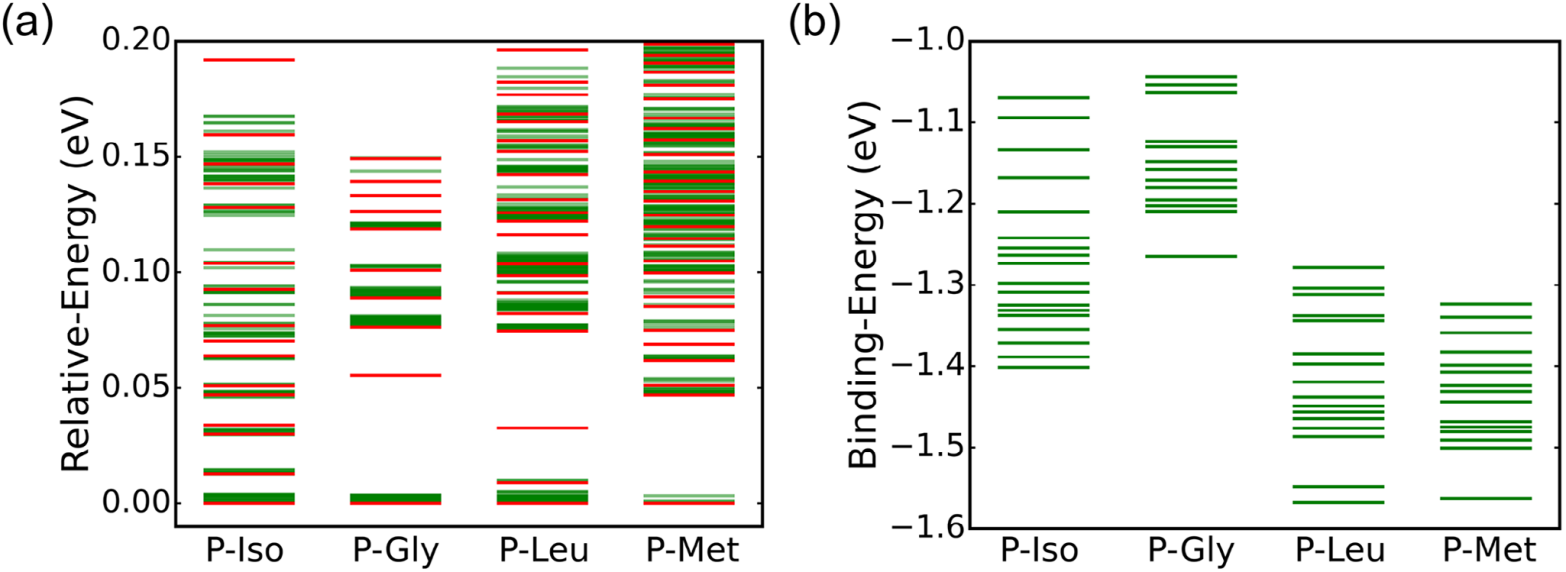
a) DFT relaxed energies (green lines) of low‐energy structures in each system and the K‐means centers (red lines) in each system. b) Binding energies of final unique structures in each system.

From the obtained PES, 400 local minima were identified for P‐Iso, 273 for P‐Gly, 365 for P‐Leu, and 317 for P‐Met. These local minima were refined using DFT calculations to ensure structural stability and eliminate unphysical configurations. Single‐point energy calculations were first performed on the identified local minimum structures using DFT, followed by the removal of unphysical configurations. The remaining structures underwent geometry optimization for 100 steps to relax their configurations. Among the relaxed structures, those with energies within 0.2 eV of the lowest‐energy conformation were selected for further analysis, with their energies represented as green lines in **Figure** **4**a. To classify the structures based on energy profiles, the K‐means clustering method was applied to determine representative cluster centers (indicated by red lines in Figure 4a), corresponding to the most optimal or typical conformations for each system. The cluster center structures were then subjected to continuous DFT optimization to ensure convergence to stable configurations. After eliminating duplicate structures, 19 unique low‐energy structures were obtained for P‐Iso, 13 for P‐Gly, 15 for P‐Leu, and 15 for P‐Met.

To describe the interaction between amino acid molecules and [PO_40_Mo_12_] ^3−^ in each system, the binding energy is E_b_ = E_P‐amino_ – E_P_ – E_amino_. Here, E_P‐amino_ is the energy of a unique structure, E_P_ is the energy of [PO_40_Mo_12_]^3−^, and E_amino_ corresponds to the energy of the respective amino acid molecules (as shown in Figure 3). A negative value indicates an exothermic adsorption process when an amino acid molecule binds to [PO_40_Mo_12_].^3−^ Furthermore, a larger absolute value of E_b_ suggests a stronger interaction between the amino acid and the cluster. The binding energies of these unique structures in each system were plotted as green lines in Figure 4b. The binding energies of amino acids on the [PO_40_Mo_12_] ^3−^ reveal distinct trends influenced by their molecular structures. P‐Iso and P‐Gly exhibit relatively narrow energy distributions (−1.1 to −1.4 eV and −1.1 to −1.3 eV), indicating stable and uniform interaction with the cluster surface. In contrast, P‐Leu and P‐Met display broader energy distributions, with binding energies ranging from −1.2 to −1.5 eV for P‐Leu and −1.3 to −1.6 eV for P‐Met. These broader distributions indicate higher conformational flexibility and stronger interactions with the cluster surface. P‐Met, in particular, exhibits the strongest adsorption strength among the four amino acids, likely due to its larger molecular size and the presence of sulfur‐containing groups that enhance binding.

**Figure 5:**
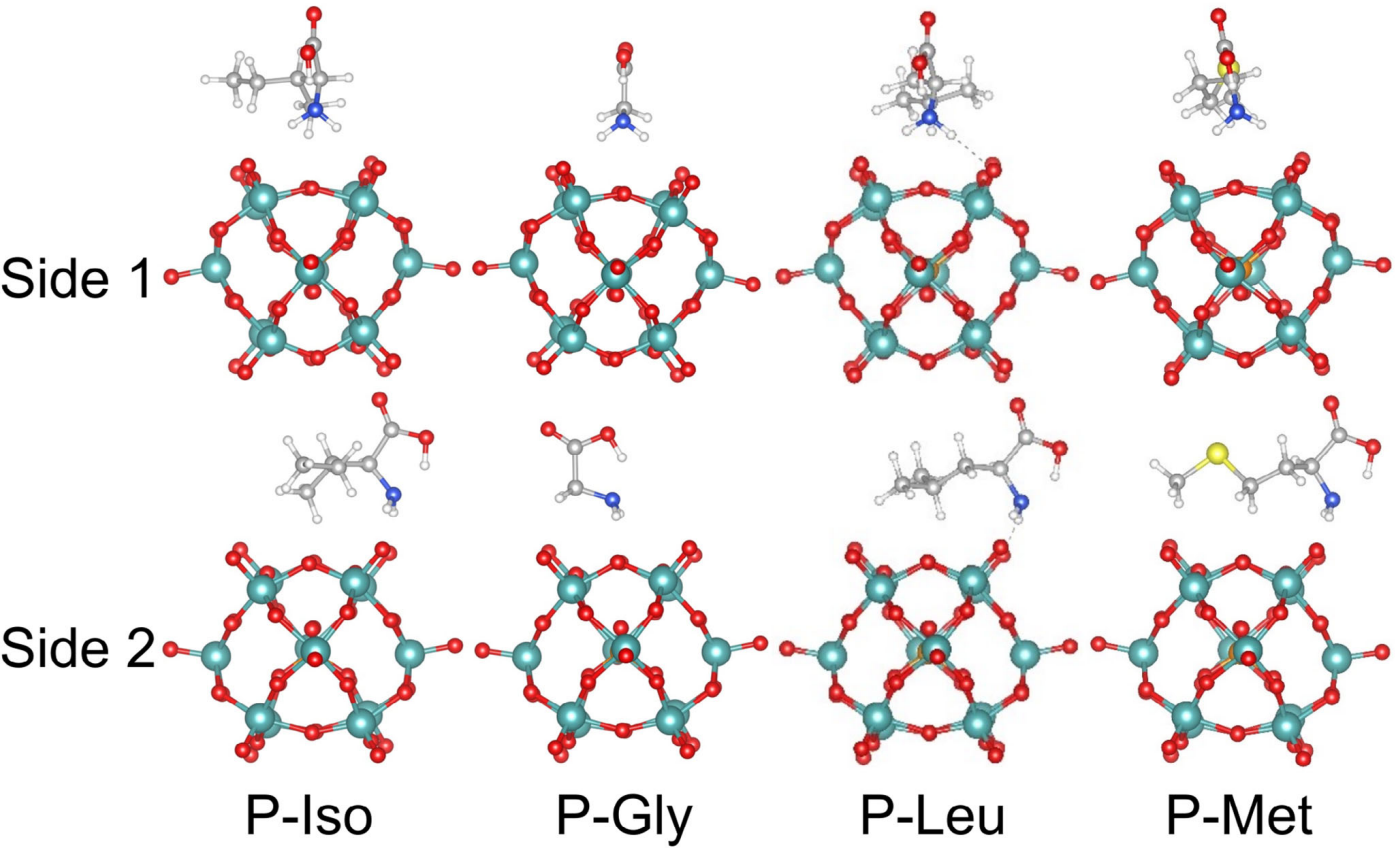
Predicted the lowest structures of P‐Iso, P‐Gly, P‐Leu, and P‐Met. There are two side views of [PO_40_Mo_12_]^3−^, an upright heart shape (Side 1) and an inverted heart shape (Side 2).

[PO_40_Mo_12_]^3−^ has a Keggin structure with T_d_ symmetry, characterized by a central P atom surrounded by 12 Mo–O octahedra. The 12 outermost oxygen atoms form a framework composed of 6 quadrilaterals and 8 triangles connected, which provides multiple binding sites for amino acid molecules. **Figure** **5** illustrates the predicted lowest energy conformer for each system. In all cases, the amino acid molecule is adsorbed on top of the quadrilateral, where the hydrogen atoms on its amino group and the CH_3_/CH_2_ group form hydrogen bonds with the oxygen atoms on the top. Furthermore, the amino acid molecule inherits the intramolecular hydrogen bonds observed in its gas phase, which is consistent with the conclusions reported in the literature. Furthermore, we found that in the P‐Met system, the flexibility of the C─S─C bond causes the terminal CH_3_ group to preferentially interact with the cluster, resulting in a deviation of the amino acid structure from its gas‐phase molecular conformation.

Figures S4–S7 (Supporting Information) present the top 10 lowest‐energy configurations predicted for all systems. For all structures, the amino group and the CH_2_/CH_3_ are positioned in closest proximity to the cluster, whereas the carboxyl group is located at a relatively greater distance. The minimal distances between the amino acids and the cluster were calculated for all configurations, revealing that the average of these minimal distances for the 10 configurations in each system is ≈2.1 Å. This indicates that hydrogen atoms on the amino acids form hydrogen bonds with the oxygen atoms on the outermost framework of the cluster. Additionally, it was observed that the adsorption of amino acids predominantly occurs above the quadrilateral in the majority of low‐energy configurations across the systems, except P‐Gly. Specifically, the distribution of adsorption positions above the quadrilateral is as follows: 7 out of 10 configurations in P‐Iso, 3 in P‐Gly, 8 in P‐Leu, and 5 in P‐Met. This suggests that amino acid molecules with more complex structures and a greater number of functional groups can adopt a wider variety of configurations upon adsorption onto the cluster surface. Furthermore, the presence of similar adsorption sites and functional groups, yet with energy distributions within 0.3 eV, implies that weak H─O interactions contribute significantly to the stabilization of the entire system.

To further investigate the stability of the predicted low‐energy structures for all adsorbate systems, we calculated the highest occupied molecular orbitals (HOMOs) and lowest unoccupied molecular orbitals (LUMOs) for all structures and obtained their HOMO‐LUMO gaps. Figure S8 (Supporting Information) depicts the HOMO and LUMO of the 10 lowest‐energy structures. The HOMO‐LUMO gaps of all low‐energy structures in systems P‐Iso, P‐Gly, and P‐Leu are ≈2.1 eV. However, for system P‐Met, the HOMO‐LUMO gaps of all low‐energy structures are below 2.1 eV, with an average gap of 1.5 eV. This may be attributed to the heavy‐atom effect associated with the sulfur atoms in P‐Met, leading to consistently lower HOMO‐LUMO gaps compared to other systems. The relatively large HOMO‐LUMO gaps indicate a low probability of electron transfer between amino acid molecules and clusters, further suggesting that all the predicted low‐energy structures in the four adsorption systems are highly stable.

We then calculated the Mülliken charges of individual atoms in the amino acid molecules for all low‐energy structures. In these systems, the average total Mülliken charge of the amino acid molecules across all low‐energy structures is ≈−0.08 e. This is due to the significant negative charge of the [PO_40_Mo_12_]^3−^ cluster (−3e), which induces Coulombic repulsion on the adsorbate amino acid molecule, making it difficult for them to acquire additional electrons. As a result, the charge variation on the adsorbed amino acid molecules remains minimal, ≈−0.08 e, without leading to a more substantial accumulation of negative charge. This indicates that all predicted low‐energy structures are highly stable across all systems and that the Coulombic repulsion between the [PO_40_Mo_12_]^3−^ and the adsorbed amino acid molecules helps to balance the hydrogen bonding interactions.

The binding energy values, ranging from −1.1 to −1.6 eV, indicate a moderate adsorption strength, suggesting weak but significant interactions between amino acid molecules and [PO_40_Mo_12_].^3−^ Furthermore, the structure analysis, HOMO‐LUMO calculations, and Mülliken charge calculations for the low‐energy structures of four systems reveal that [PO_40_Mo_12_]^3−^ and amino acid‐adsorbed systems are stabilized by the physical interaction between H atoms within the molecule and O atoms in the [PO_40_Mo_12_].^3−^ It can be inferred that [PO_40_Mo_12_]^3−^ can strongly bind with molecular groups in Aβ peptide to modulate aggregation but without destroying any bio‐molecules (like other protein chains and functional organic molecules).

### 2.3 [PO_40_Mo_12_]^3−^‐Peptide‐Chain Interaction Systems

Proteins are characterized by a large number of outer molecular groups surrounding the peptide bonds, including ─CH_3_, ─CH_2_CH_3_, ─CONH_2_, ─C_6_H_5_, ─CHC_3_H_3_N_2_H_2_, ─COO, etc. To further investigate the microscopic mechanism for clusters [PO_40_Mo_12_]^3−^ that inhibit protein aggregation or fibrillization, we study the microscopic structures between [PO_40_Mo_12_]^3−^ and the molecular groups in Aβ.

However, due to the large number of atoms in an entire protein molecule, it is challenging to perform simulations using the all‐electron code FHI‐aims or other DFT codes. Thus, we extract a segment of the peptide chain to construct the adsorption system with [PO_40_Mo_12_].^3−^ Here, the peptide chain is kept fixed as a chain shape, while the search space is defined based on the mass center (x_2_, y_2_, and z_2_) of [PO_40_Mo_12_]^3−^ and its rotational degrees of freedom (h_2_, i_2_, and j_2_), as shown in Figure 2c. Subsequently, the PES was actively learned using the BOSS to obtain low‐energy structures.

**Figure 6:**
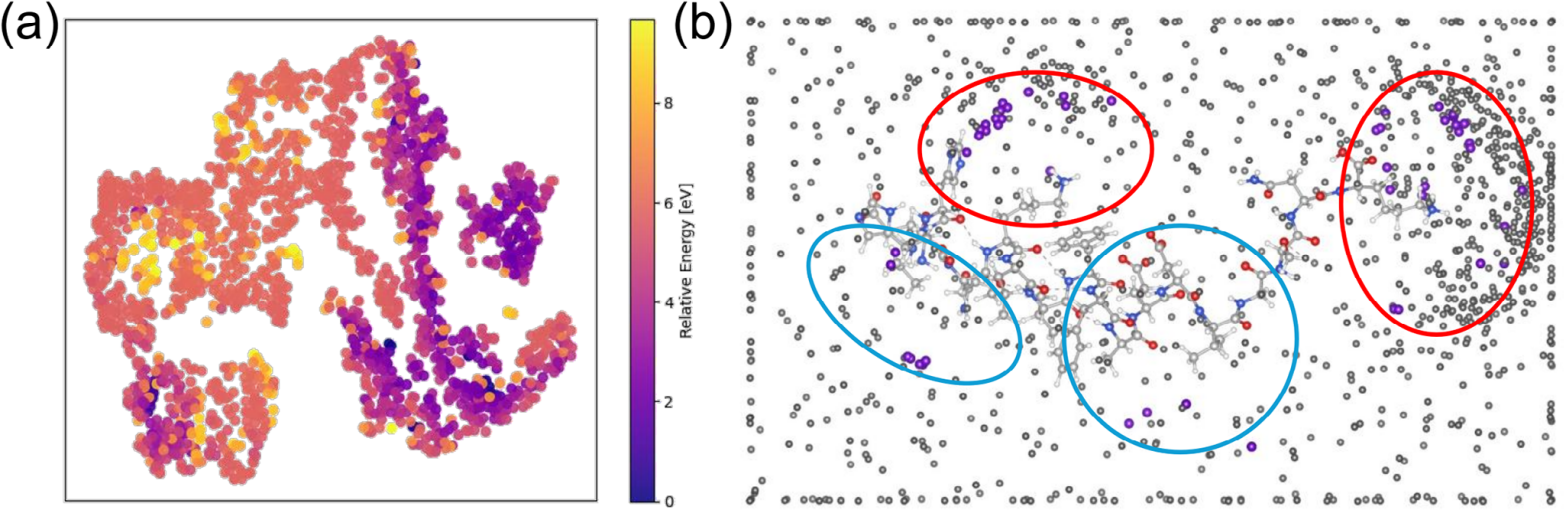
a) The 2D space of t‐SNE dimensionality reduction for cluster centers obtained through BOSS sampling. Each point represents a sampled structure, with different colors indicating its corresponding energy. b) BOSS sampled 1200 mass centers (gray dots) of [PO_40_Mo_12_]^3−^ in 3D space. Fifty‐three mass centers (purple dots) of [PO_40_Mo_12_]^3−^ in final local minimum structures are distributed in 3D space.

BOSS sampled 1200 configurations to construct a surrogate GP model for the PES simulation. The high‐dimensional structural features (center position of [PO_40_Mo_12_]^3−^ in each configuration) of these configurations were projected into a 2D latent space through t‐distributed stochastic neighbor embedding (t‐SNE), as shown in Fi**Figure** **6**a. Each data point's spatial coordinates correspond to a specific configuration's position in phase space, with color intensity following an energy scale: deep purple marks low‐energy stable configurations (∆E ≈ 0.0 eV), while bright yellow indicates high‐energy configurations (∆E > 9.6 eV). The topological reconstruction reveals discrete potential well basins interconnected by transition channels within the configuration space, where isoenergetic configurations self‐organize into distinct clusters demonstrating strong structure‐energy correspondence. This mapping quantitatively validates the BOSS code's dual exploration capability across both low‐energy minima and high‐energy transition states on the PES. In addition, Figure 6a shows that the distribution of high‐energy structures (2 eV < E < 6 eV) covers the distribution of low‐energy structures (E < 2 eV). This shows that BOSS is accurate and efficient in sampling the entire phase space when constructing the PES. Here, E is the single‐point energy of each sampling configuration.

To directly visualize the center of mass (x_2_, y_2_, z_2_) distribution of the 1200 sampled structures, a 3D spatial mapping incorporating both the peptide chain coordinates and the mass center positions was constructed as shown in Figure 6b. Analysis results revealed an inverse correlation between oxygen atom density and sampling frequency ‐regions with higher oxygen content exhibited significantly lower sampling rates. Because BOSS tends to deeply dig around the local minimum region in the search space, this observation led to the identification of two distinct low‐energy regions (highlighted by red circles in Figure 6b), which show remarkable consistency with the t‐SNE dimensionality reduction results revealing two prominent low‐energy clusters in Figure 6a.

From the 587 low‐energy points identified on the BOSS‐predicted PES, we implemented a multi‐stage filtering process: 1) removal of non‐physical configurations, 2) elimination of duplicate structures, and 3) exclusion of systems where amino acid residues were beyond practical interaction distances from the [PO_40_Mo_12_]^3−^ cluster. This rigorous selection yielded 53 viable adsorption structures for subsequent DFT relaxation. Their spatial distribution, marked by purple dots in Figure 6b, reveals that 40 structures occupy the two primary low‐energy regions (marked by two red circles), while a minority populate a secondary low‐energy zone (marked by two blue circles). Notably, no stable configurations were found in the oxygen‐exposed areas (central peptide chain segment).

**Figure 7:**
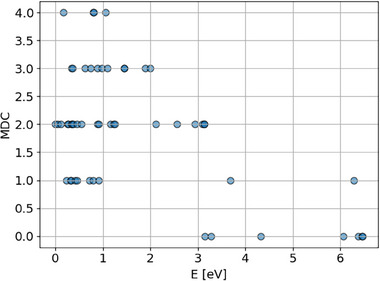
The MDC versus E of 53 local minimum structures.

To investigate the interactions between the peptide chain and the [PO_40_Mo_12_]^3−^ cluster, the atomic distance matrix between the peptide and the cluster was calculated for each low‐energy structure. The number of matrix elements with distances less than 2.2 Å, representing close contacts between atoms of the cluster and the peptide, was then counted. This count, referred to as the minimum distance counts (MDC), quantifies the extent of weak interactions in each structure. **Figure** **7** presents the relationship between MDC and the corresponding structure energies (E) for 53 low‐energy structures. A generally negative correlation is observed, indicating that structures with more weakly interacting atomic pairs tend to have lower energies. Notably, approximately 70% of the structures exhibit at least two such interactions, with some containing as many as three or four.

Combined with spatial analysis, we infer that most structures involve multiple interactions between H (from the peptide chain) and O (from [PO_40_Mo_12_]^3−^). Furthermore, compared to the molecular group highlighted in the left red circle of Figure 6b, the chain‐like structure of the CH_2_CH_2_CH_2_CH_2_CH_3_ group (in the right red circle of Figure 6b) appears more favorable for forming multiple hydrogen‐bond networks with the cluster. This enhanced interaction contributes to increased structural stability and lower overall energy. The Mülliken charge analysis and charge difference density of low‐energy structures in SI (Table S1 and Figure S11, Supporting Information) also support these points.This interaction pattern is associated with significantly lower binding energies, approximately 5 eV, in the corresponding regions (Figure S9, Supporting Information). Energy comparisons reveal notable differences: a variation of ≈1 eV between structures within the two primary low‐energy regions, and differences exceeding 3 eV between the primary (red) and secondary (blue) regions. Figure S10 (Supporting Information) depicts the ball‐and‐stick representations of 6 low‐energy structures.

### 2.4 Advantages and Limitations of Methods

Compared to conventional exhaustive, stochastic, or systematic search methods, this methods offer several distinct advantages, especially for high‐dimensional systems such as the POM–Aβ interface. First, BOSS's active learning strategy significantly reduces the number of expensive DFT evaluations required by efficiently guiding sampling toward the most informative regions of the potential energy surface (PES). For example, in this study, BOSS was able to accurately fit the PES of each system (5‐6 dimension) by sampling and evaluating the energies of only 1200 structures. Other methods, like systematic methods or random algorithms, may need 100 000 or 1 000 000 sampling points. Second, BOSS is particularly well‐suited for identifying multiple low‐energy minima in complex, high‐dimensional landscapes, a critical requirement for capturing the flexible and heterogeneous nature of biomolecular adsorption. For [PO_40_Mo_12_]^3−^‐peptide‐chain interaction systems, our methods accurately and efficiently identified 53 low‐energy structures. This demonstrates that our methods can not only obtain global and local minima efficiently, but also reveal the diversity of relevant interaction modes within a feasible computational cost, which is difficult to achieve with traditional methods. Third, in systems like ours, where the configurational space is vast due to flexible peptide backbones and multiple possible binding sites, the computational cost of traditional brute‐force or random sampling approaches becomes intractable, while BOSS maintains both scalability and precision. These advantages collectively make BOSS a powerful and practical tool for studying hybrid bio‐inorganic systems at the atomic level, where chemical complexity and structural variability present formidable challenges to standard methods.

While the method demonstrates notable advantages in efficiently and accurately exploring high‐dimensional PES and identifying multiple low‐energy binding configurations, it is equally important to recognize the limitations of our current modeling approach. First, the DFT calculations were performed in the gas phase, without incorporating explicit solvent effects or physiological ionic strength, which may affect interaction energies and structural stability under biological conditions. Second, during the BOSS‐based structure search, the peptide backbone was constrained to reduce computational complexity, thus limiting the sampling of large‐scale conformational changes that may occur in vivo. Third, the choice of DFT functional and basis set may introduce systematic deviations in the calculated binding energies. Finally, and most importantly, the biological relevance of the predicted binding configurations remains to be validated by experiments. Despite these limitations, our study provides a valuable theoretical framework for guiding future experimental investigations into POM–Aβ interactions.

## 3 Conclusion

In this study, we combined active‐learning BO with the DFT method to systematically investigate the interaction mechanisms between [PO_40_Mo_12_]^3−^ and biomolecules. Specially, it can be reasonably inferred that negatively charged [PO_40_Mo_12_]^3−^ tends to engage in non‐covalent physical adsorption with other amino acid molecules and Aβ peptides. Importantly, the negligible charge transfer between [PO_40_Mo_12_]^3−^ and amino acid molecules suggests that the cluster does not induce significant electronic perturbations, thereby supporting its potential as a non‐toxic therapeutic agent for AD. Simulation of [PO_40_Mo_12_]^3−^‐peptide‐chain adsorption system reveals that [PO_40_Mo_12_]^3−^ preferentially binds to oxygen‐deficient single‐branched molecular groups via multisite interactions. Significant energy differences (≥ 1 – 3 eV) between various binding configurations result in deep potential wells, which in turn promote strong physical interactions between the cluster and the peptide chain, which may be limiting Aβ aggregation. In addition, H‐bond formation in the local minimum structures occurs at multiple peptide sites, not limited to specific functional groups, which suggests that the synergistic combination of multiple non‐covalent weak interactions may enable strong binding between [PO_40_Mo_12_]^3−^ clusters and aggregate Aβ. Crucially, the absence of covalent bonding or chemical interactions ensures these interactions maintain biological fidelity—a key therapeutic advantage that minimizes side effects for AD treatment.

Overall, this study can provide theoretical evidence that [PO_40_Mo_12_]^3−^ may serve as an effective Aβ aggregation inhibitor by binding with multi‐H atoms in ─CH_n_ of side chains (alkyl chains). Importantly, the absence of chemical reactions between [PO_40_Mo_12_]^3−^ and Aβ ensures low side effects and low toxicity for patients. For Aβ, four amino acids (valine, lysine, leucine, and isoleucine) contain alkyl chains in the corresponding positions (12, 16, 17, 18, 24, 28, 31, 32, 34, 36, 39, 40, and 41), which means that POMs or other nanoparticles with negative charges are easily form non‐chemical interactions with Aβ chain through binding amino acid side chains at the above positions.

As a computational study, our conclusions are theoretical in nature and await experimental validation. We recommend future investigations using cryo‐EM, NMR spectroscopy, or site‐directed mutagenesis—such as alanine scanning of Val12, Leu17, and Ile31—to test the predicted binding modes and mechanisms. These efforts would provide critical support for the therapeutic potential of POMs and further clarify their role in modulating Aβ aggregation.

Beyond identifying potential binding modes, our findings also offer theoretical guidance for the rational design of more effective POM‐based therapeutic agents. Specifically, favoring POM architectures that maximize the number of accessible surface oxygen atoms could promote multivalent hydrogen bonding and hydrophobic interactions. In addition, tuning the size and symmetry of POM clusters to match the spatial arrangement of hydrophobic residues on Aβ peptides, particularly Val12, Leu17, and Ile31, may enhance binding specificity. Furthermore, moderating the overall negative charge of POMs may help optimize the balance between binding affinity and minimizing electrostatic repulsion or unintended interactions with off‐target biomolecules. These design principles, derived from our computational framework, provide a starting point for future molecular engineering. We also emphasize that experimental validation, such as site‐directed mutagenesis and high‐resolution structural techniques (e.g., cryo‐EM or NMR), will be essential to confirm and refine these theoretical insights.

## Conflict of Interest

The authors declare no conflict of interest.

## Supporting information



Supporting Information

## Data Availability

The data that support the findings of this study are available from the corresponding author upon reasonable request.
