# Supplementary material for: Active Learning‐Assisted Exploration of [PO40Mo12]3− for Alzheimer's Therapy Insights
Source: Adv Sci (Weinh). 2025 Aug 19;12(42):e08702. doi: 10.1002/advs.202508702 (PMC12622557; doi:10.1002/advs.202508702)
Supplement: Supplementary file 1 — Supporting Information [file ADVS-12-e08702-s001.docx]

Supplementary Material

Active Learning-Assisted Exploration of [PO_40_Mo_12_]^3−^ for Alzheimer’s Therapy Insights

Lincan Fang*, Ruoxue Peng, Luping Xia, and Guilin Zhuang*

*School of Chemistry and Materials Science, Anhui Normal University, Wuhu, Anhui, 241002, China*

E-mail: [glzhuang@ahnu.edu.cn](mailto:glzhuang@ahnu.edu.cn)； lincan.fang@ahnu.edu.cn;

**Figure of Contents**

S1: Ball-stick model and defined search space of (a) Iso, (b) Leu, and (c) Met.

S2: DFT single-point energies for the BOSS-predicted local minimum structures for four amino acid molecules.

S3: Ball-stick model and defined search space of (a) P-Gly, (b) P-Leu, and (c) P-Met. S4: The 10 lowest-energy structures predicted by BOSS for P-Iso.

S5: The 10 lowest-energy structures predicted by BOSS for P-Gly. S6: The 10 lowest-energy structures predicted by BOSS for P-Leu. S7: The 10 lowest-energy structures predicted by BOSS for P-Met.

S8: The HOMO-LUMO of all the predicted top 10 lowest-energy structures of P-Iso (red lines), P-Gly (blue lines), P-Leu (orange lines), and P-Met (black lines).

Table S1: The binding energies (eV) of top 10 stable low-energy structures for each system.

S9: The binding energy of 53 BOSS predicted low-energy structures of the [PO_40_Mo_12_]^3−^-peptide-chain adsorption system.

S10: The ball-and-stick representations of 6 low-energy structures for the [PO_40_Mo_12_]^3−^-peptide-chain adsorption system.

Figure S11: The peptide charge of low-energy structures for the [PO_40_Mo_12_]^3−^-peptide-chain adsorption system.

Figure S12: The charge difference density of four low-energy structures for the [PO_40_Mo_12_]^3−^-peptide-chain adsorption system.

Figure S13: Overview of BOSS-based procedure for conformer search, featuring (i) system preparation, (ii) BOSS active learn PES, and (iii) DFT relax local minimum structures.

Mülliken charges and Charge Density Difference

Methods parts: Keywords setting for FHI-aims and BOSS.


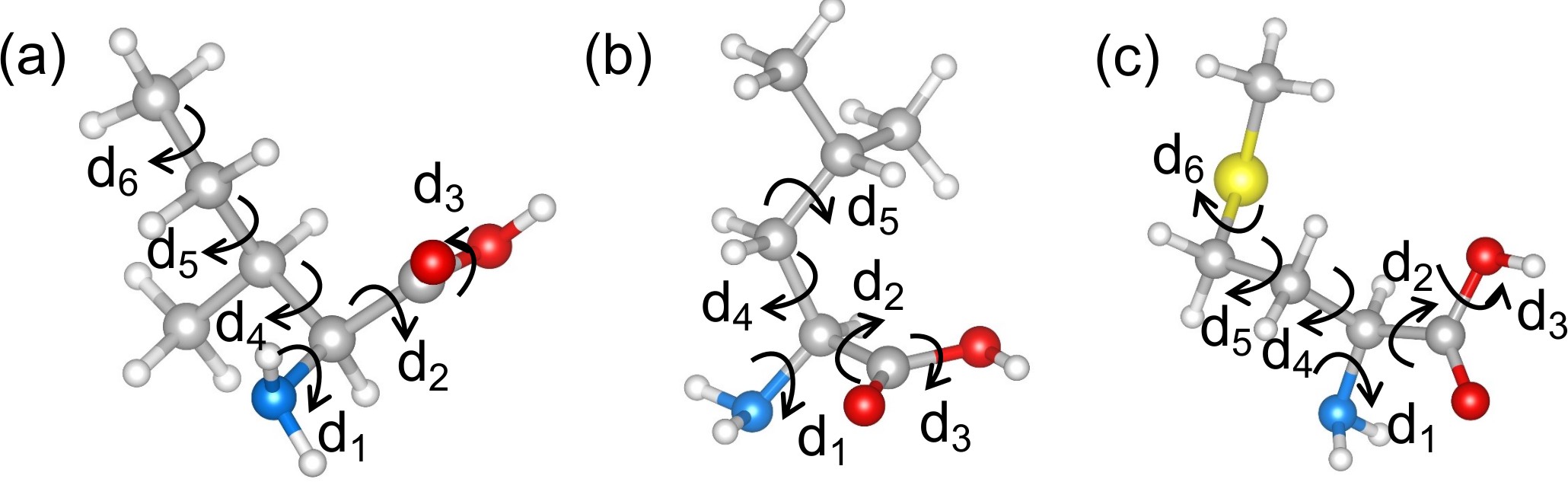


Figure S1: Ball-stick model and defined search space of (a) Iso, (b) Leu, and (c) Met.


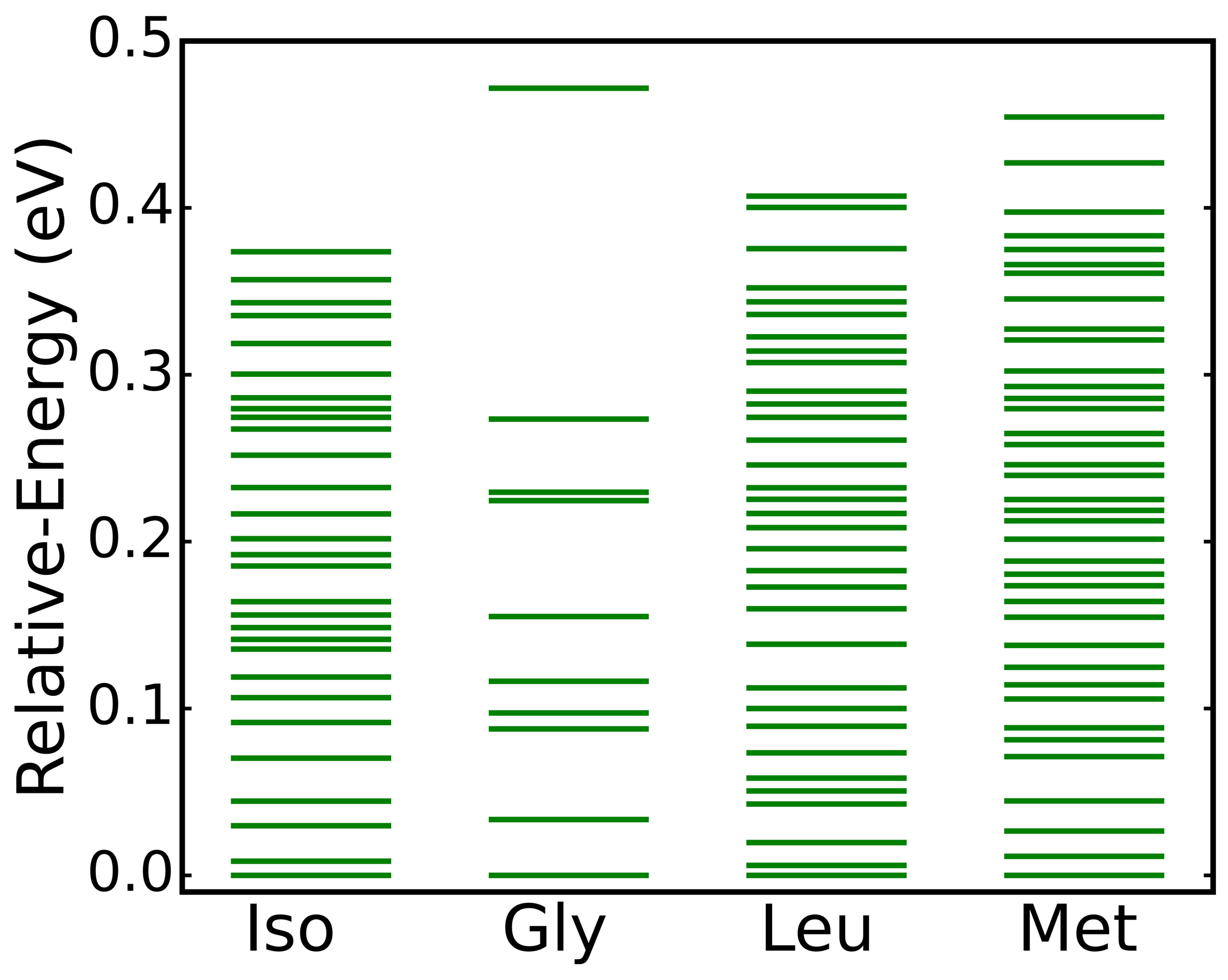


Figure S2: DFT single-point energies for the BOSS-predicted local minimum structures of Iso, Gly, Leu, and Met.


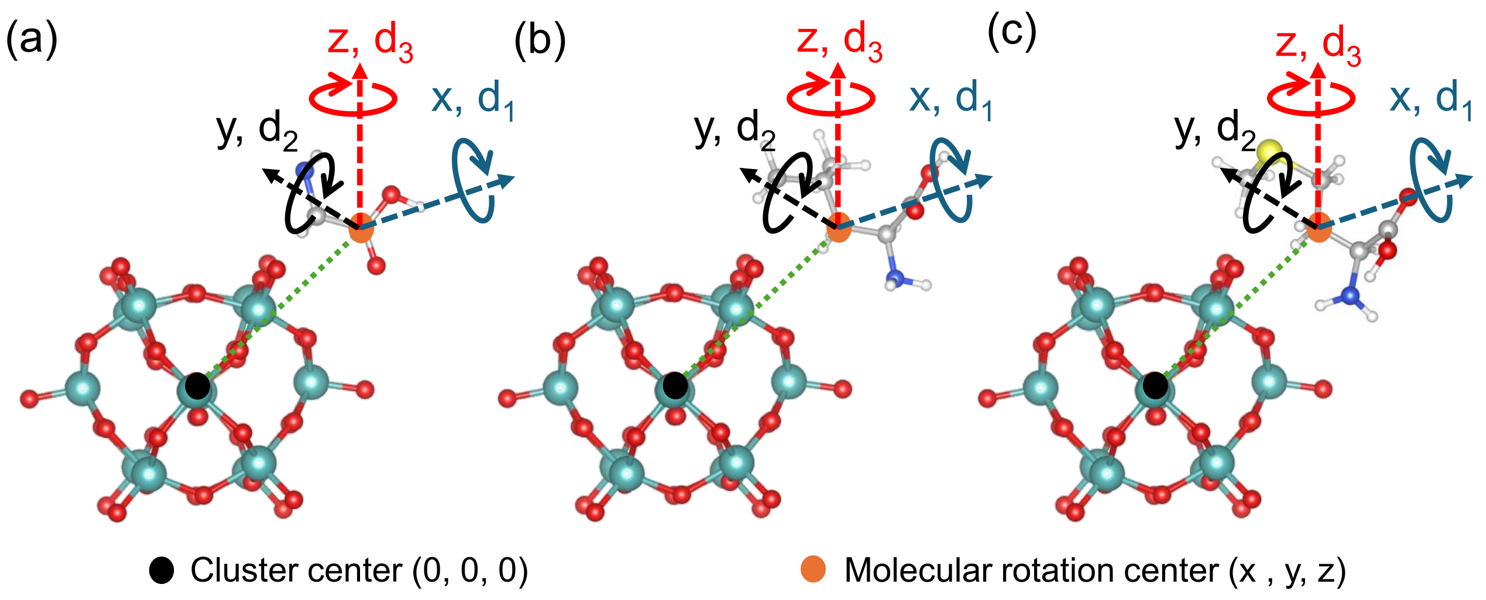


Figure S3: Ball-stick model and defined search space of (a) P-Gly, (b) P-Leu, and (c) P-Met


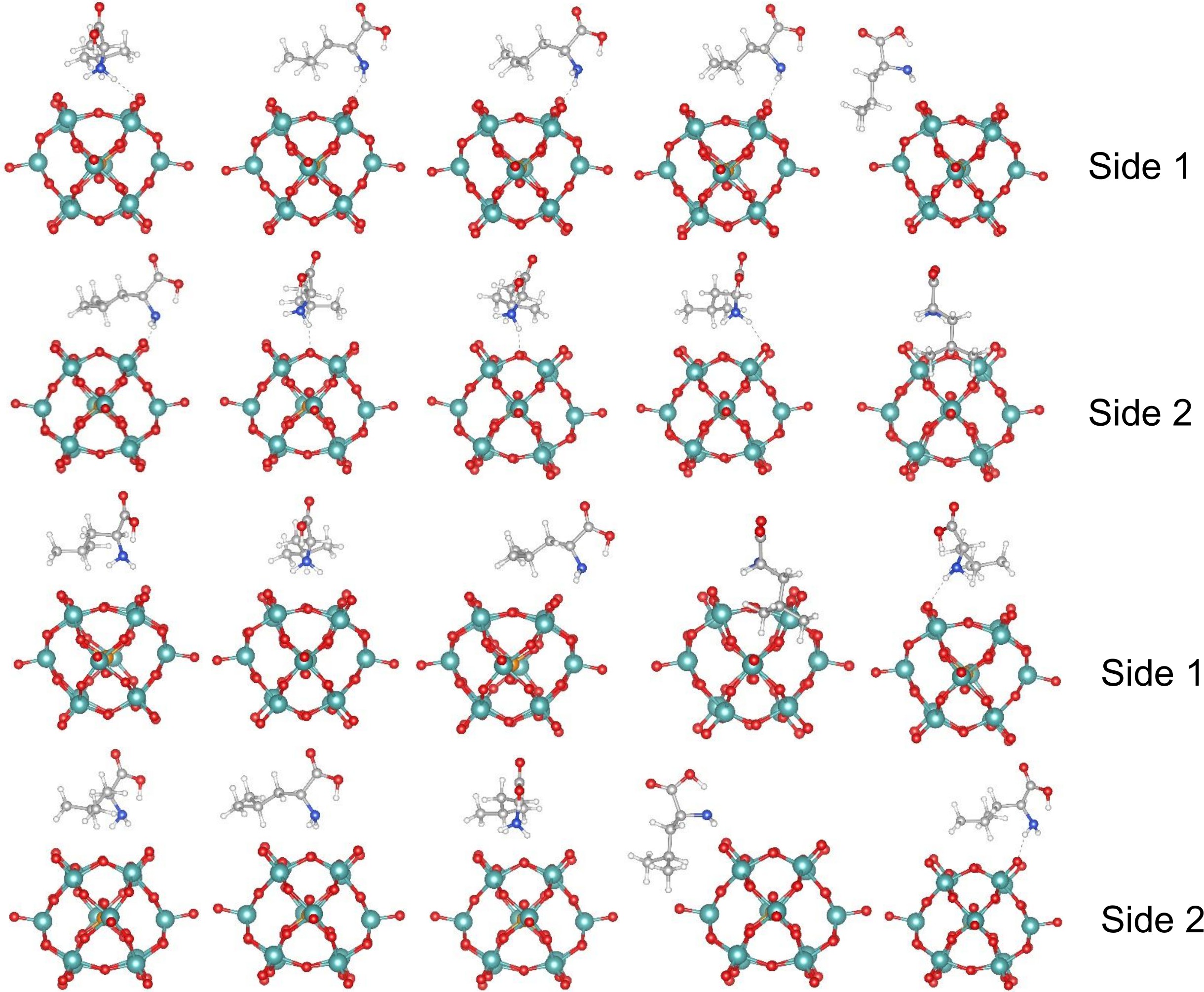


Figure S4: The 10 lowest-energy structures predicted by BOSS for P-Iso.


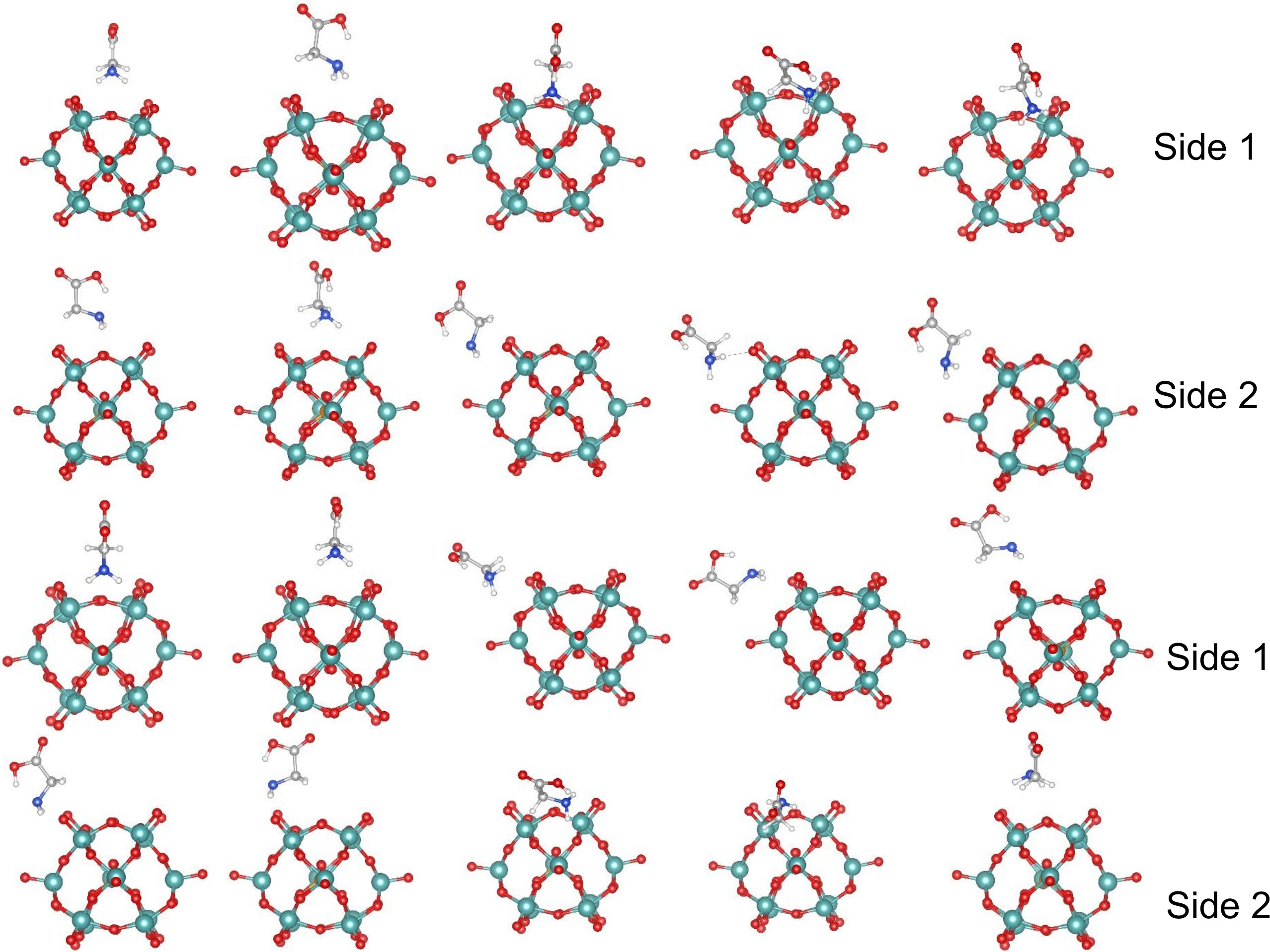


Figure S5: The 10 lowest-energy structures predicted by BOSS for P-Gly.


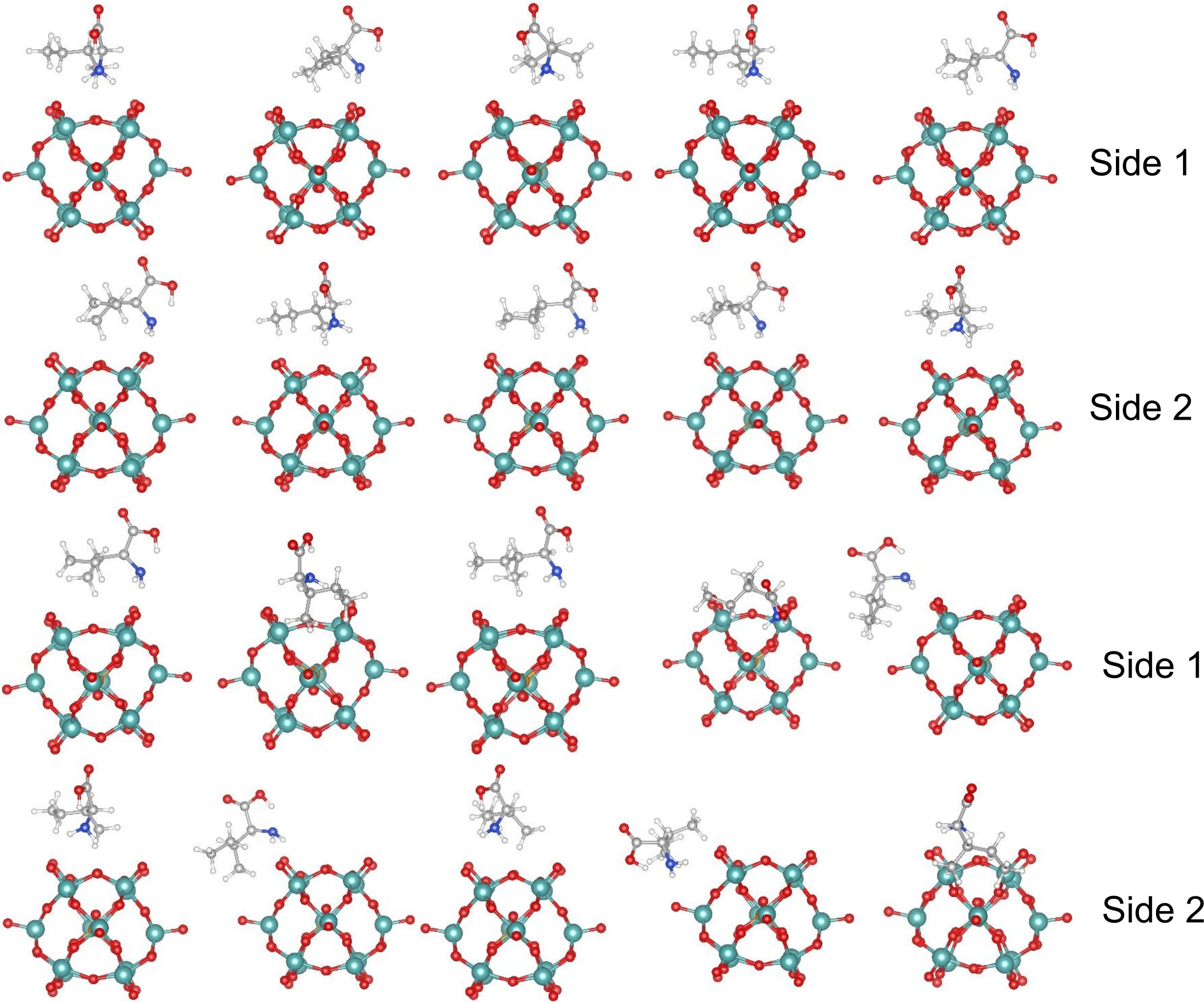


Figure S6: The 10 lowest-energy structures predicted by BOSS for P-Leu.


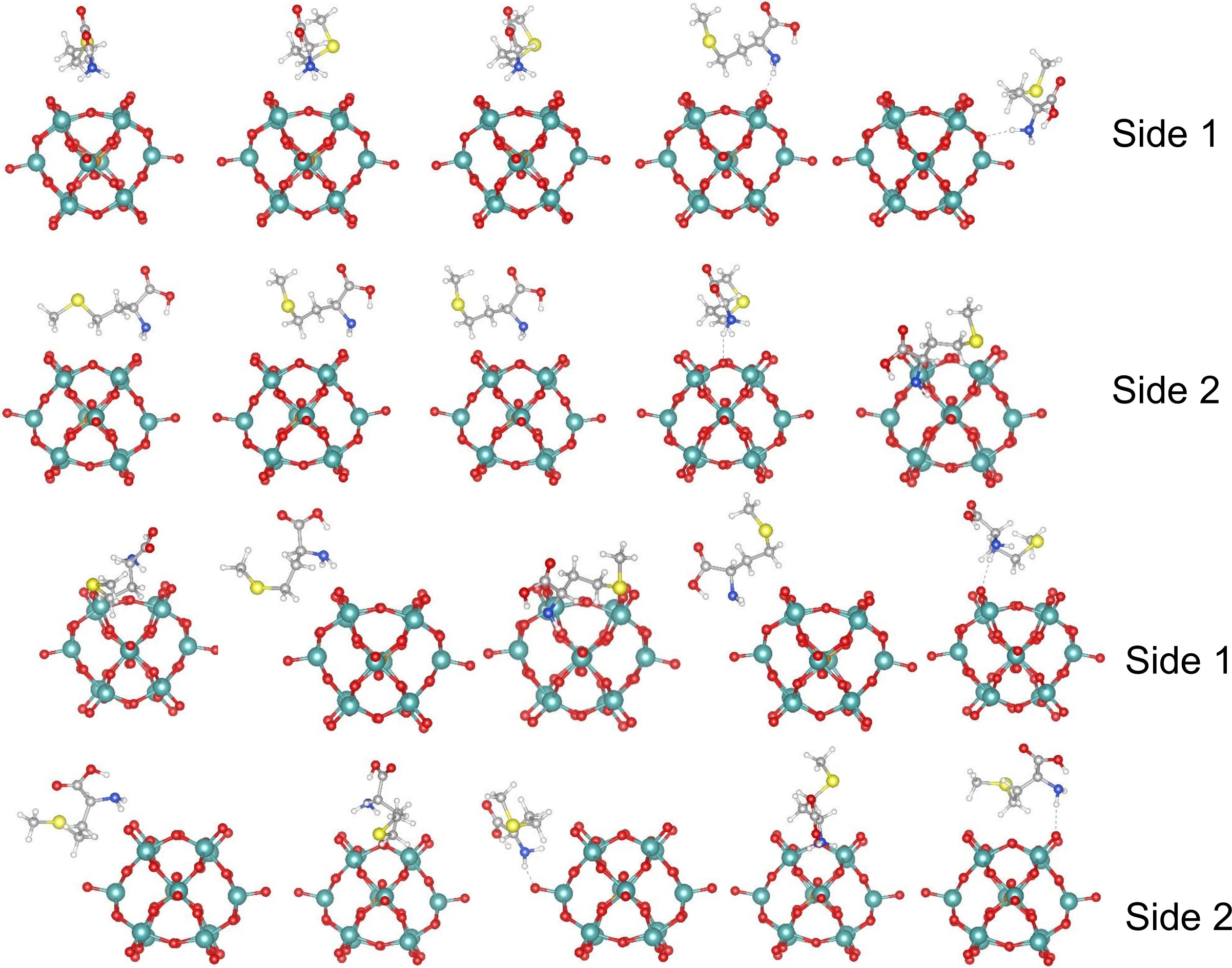


Figure S7: The 10 lowest-energy structures predicted by BOSS for P-Met.


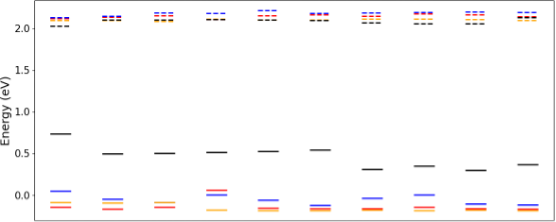


Figure S8: The HOMO (solid lines) and LUMO (dashed lines) of all the predicted top 10 lowest-energy structures in P-Iso (red lines), P-Gly (blue lines), P-Leu (orange lines), and P-Met (black lines).

| P-Iso |  | -1.401 | -1.389 | -1.372 | -1.354 | -1.338 | -1.331 | -1.324 | -1.309 | -1.298 | -1.273 |
| --- | --- | --- | --- | --- | --- | --- | --- | --- | --- | --- | --- |
| P-Gly |  | -1.264 | -1.210 | -1.202 | -1.195 | -1.180 | -1.701 | -1.158 | -1.148 | -1.130 | -1.123 |
| P-Leu |  | -1.568 | -1.549 | -1.487 | -1.476 | -1.464 | -1.456 | -1.449 | -1.438 | -1.419 | -1.397 |
| P-Met |  | -1.562 | -1.501 | -1.491 | -1.481 | -1.475 | -1.468 | -1.443 | -1.431 | -1.432 | -1.407 |

Table S1: The binding energies (eV) of top 10 stable low-energy structures for each system.


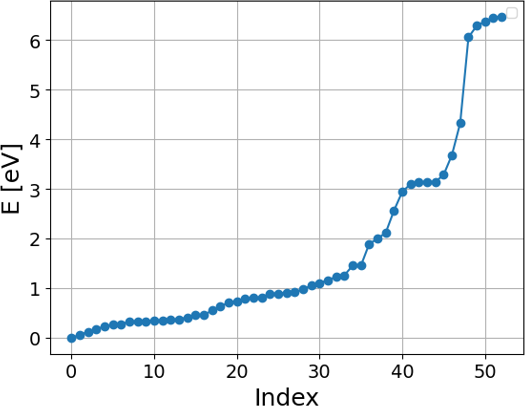


Figure S9: The energy of 58 BOSS predicted low-energy structures of the [PO_40_Mo_12_]^3^*^−^*- peptide-chain adsorption system.


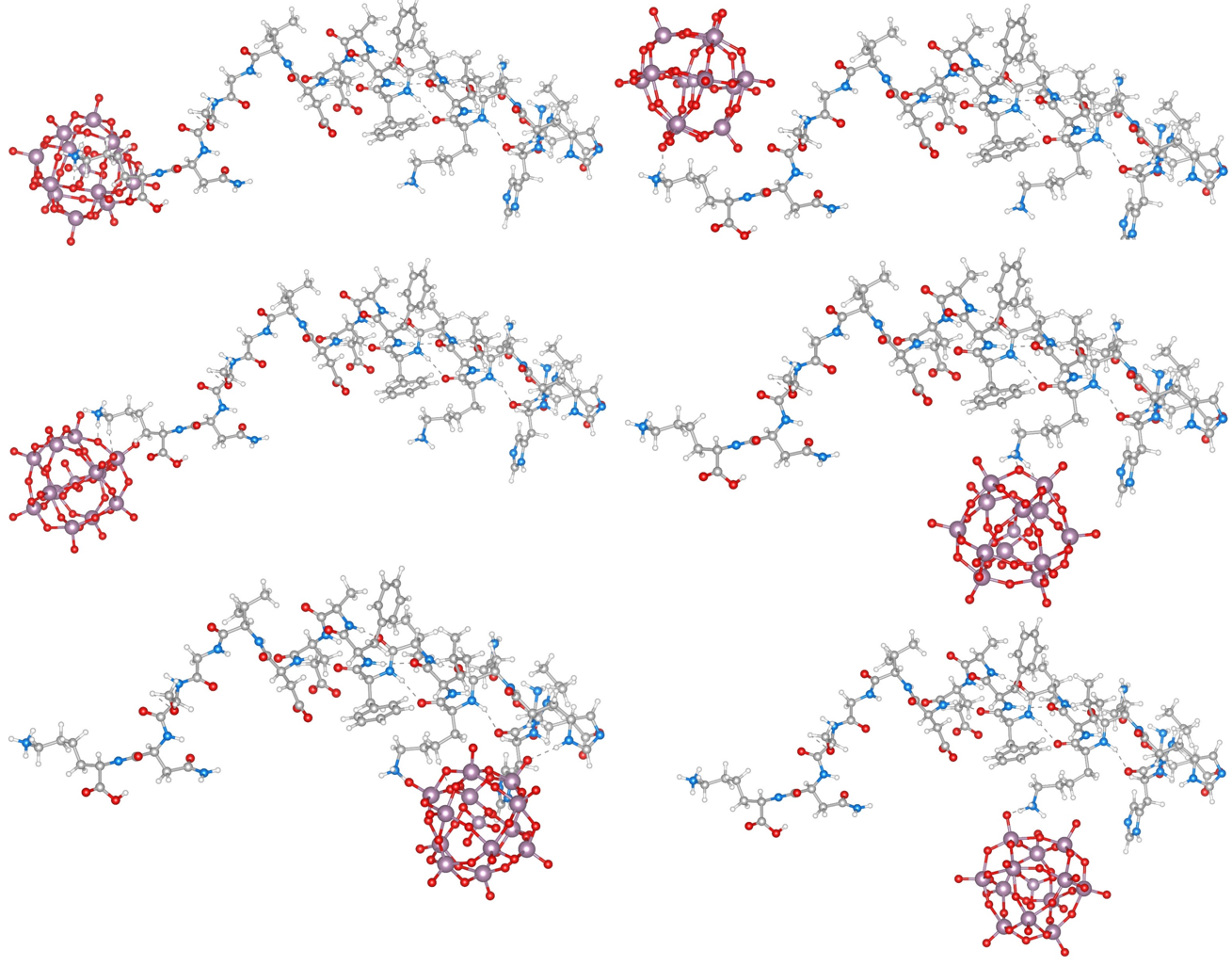


Figure S10: The ball-and-stick representations of 6 low-energy structures for the [PO_40_Mo_12_]^3−^-peptide-chain adsorption system.

**Mülliken charges and Charge Density Difference**


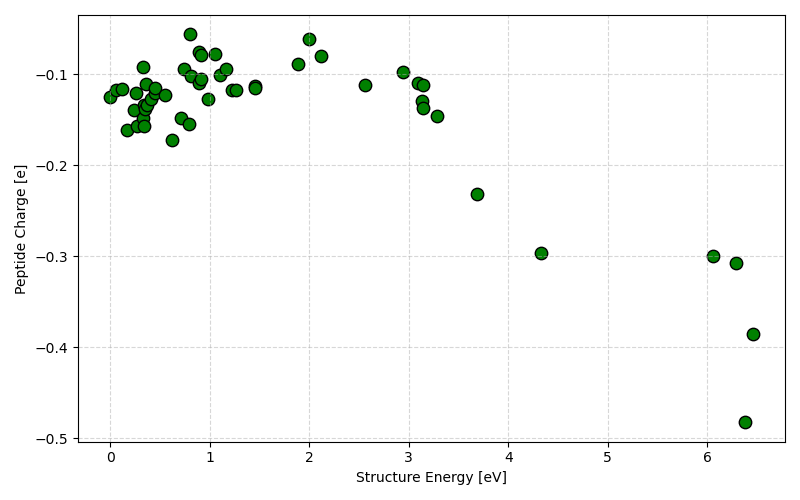


Figure S11: The peptide charge of low-energy structures for the [PO_40_Mo_12_]^3−^-peptide-chain adsorption system.


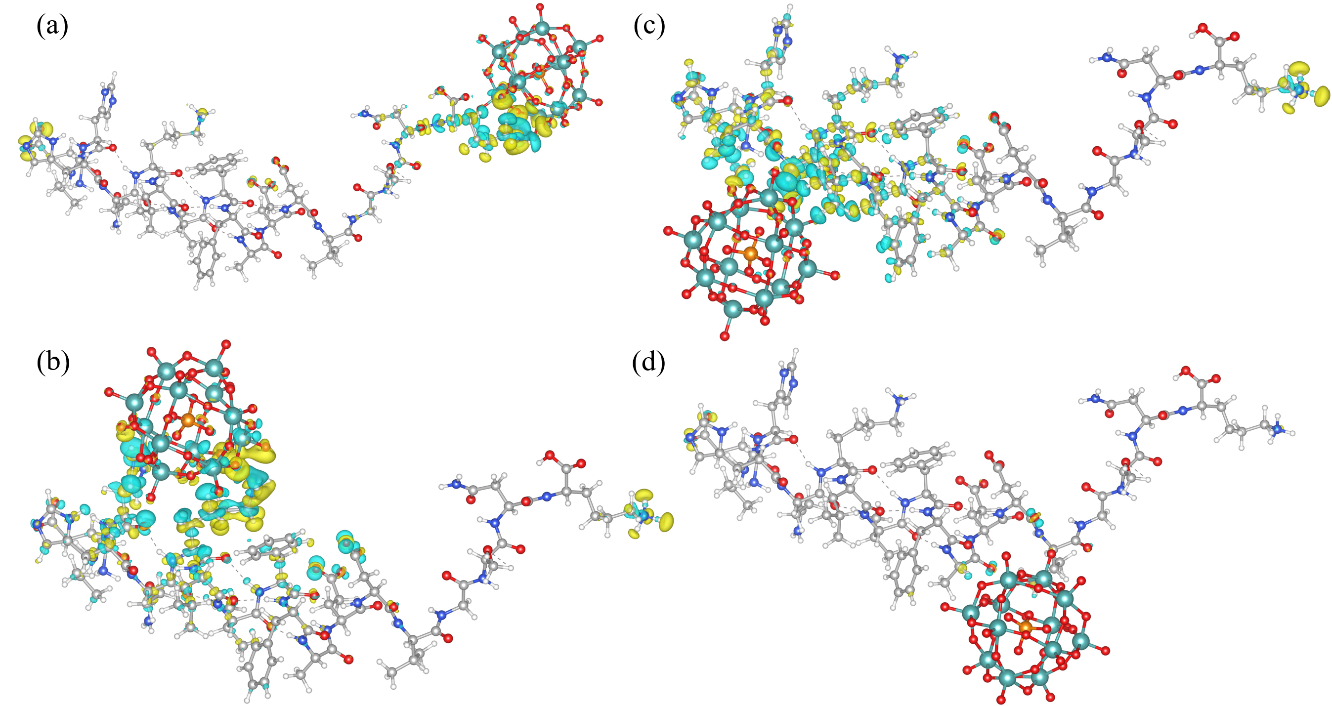


Figure S12: The charge difference density of four low-energy structures for the [PO_40_Mo_12_]^3−^-peptide-chain adsorption system. The [PO_40_Mo_12_]^3−^ position in (a) and (b) from red circle region of Figure 6(b), for (c) and (d) from blue cirle of Figure 6(b). The isosurface is 0.0015.

In this work, we calculated the Mülliken charges of individual atoms in the amino acid molecules for all low-energy structures in the [PO_40_Mo_12_]^3−^-peptide-chain adsorption system. For the structures with high energies, the total Mülliken charges of the peptide is from -0.5 to -0.2 e. Figure 7 depicts that these structures with high energies have no hydrogen bonding interactions between peptide molecule and cluster. This may be caused by the -3 e charge setting in FHI-aims simulations. For the most structrues with energies below 3.5 eV (with multiple hydrogen bond network), the total Mülliken charges of the peptide in these structures is approximately from -0.2 to -0.1 e. This indicates that significat negative charge of cluster (-3 e) induces Counlombic repulsion on the adsorbate peptide, which suggests that Counlombic repulsion and hydrogen bond network interaction stabilized structures. Additionally, the peptide charge indicates that charge transformation from peptide to cluster for most structures with hrdrogen bond network, and the charge density difference in Figure S12 also support this points.

**Computational Details**

**Keyword setting of FHI-aims**

In this work, we employed the all-electron code FHI-aims for all DFT calculations^1–3^. "Tight" numerical settings, "tier 2" basis sets for S, C, O, N, H, and Mo, and PBE+TS functional were used throughout^4,5^. For geometry optimizations, the geometry was considered to be converged when the maximum residual force was below 0.05 eV/Å.

**Keyword setting of BOSS**

In this work, BOSS samples all the dihedral angles and rotational degrees of freedom in a full range of 0^o^ to 360^o^, employing the "stpd" kernel for these periodic variables. For BOSS sampling the variables of the molecule or [PO_40_Mo_12_]^3-^ center, the sampling range is set to be 5 Å smaller than the minimum [PO_40_Mo_12_]^3-^/molecular coordinate and 5 Å larger than the maximum coordinate. The "rbf" kernel is used for these non-periodic variables. During surrogate PES model simulation, BOSS samples nonphysical structures with atomic clashes. We utilized the strategy in Ref. 6 to address this problem^6^. Gaussian process models were employed for BOSS sampling to fit surrogate PES for all systems. The exploratory confidence bound (eLCB) acquisition function was minimized to refine the GP model by acquiring more data points in configuration space.

**Usage of BOSS**

In this study, we used the structure search procedure based on BOSS (Ref: Fang L, et al. *J. Chem. Theory. Comput*., 2021, 17, 1955-1966.) to identify global and local minimum structures for each system. The procedure includes four steps: (i) System Preparation, (ii) Bayesian optimization conformer search, (iii) refinement, and (iv) validation. Since this study is a pure theoretical study, only the first three steps were carried out. Here, we use the structure search for an amino acid (Met) molecule adsorbed on [PMo_12_O_40_]³⁻ as an example to demonstrate the usage of the procedure, as shown in Figure S13.

In Step (i), the xyz-coordinates of Met molecule and [PMo_12_O_40_]³⁻ were obtained from the drug bank online database and previous studies. After building a adsorbed structure of Met on [PMo_12_O_40_]³⁻, we then employed DFT method initially to optimize the structure. The [PMo_12_O_40_]³⁻ cluster center was obtained, and the whole relaxed structure was translated so that the [PMo_12_O_40_]³⁻ cluster center is positioned at the origin (0, 0, 0). For the system of Met on [PMo_12_O_40_]³⁻, the most informative degree of freedom for describe the different conformers is the translational position of Met in space and its rotational orientation around its own center. Hence, we select Met center position and rotational degree of Met around its center to define a six dimensional search space (x, y, z, d_1_, d_2_, and d_3_ in Figure S13).

In step (ii), BOSS was employed to active sample the search space defined in Step (i) to learn a PES. In this process, only x, y, z, d_1_, d_2_, and d_3_ were sampled, and other degrees of freedom were keep fixed at their DFT-optimized value.

After the BOSS-predicted PES converged, in Step (iii), the PES was analyzed and the local minimum locations and related structures were extracted. Then, DFT was employed to relax these structures and obtain optimized structures and energies.


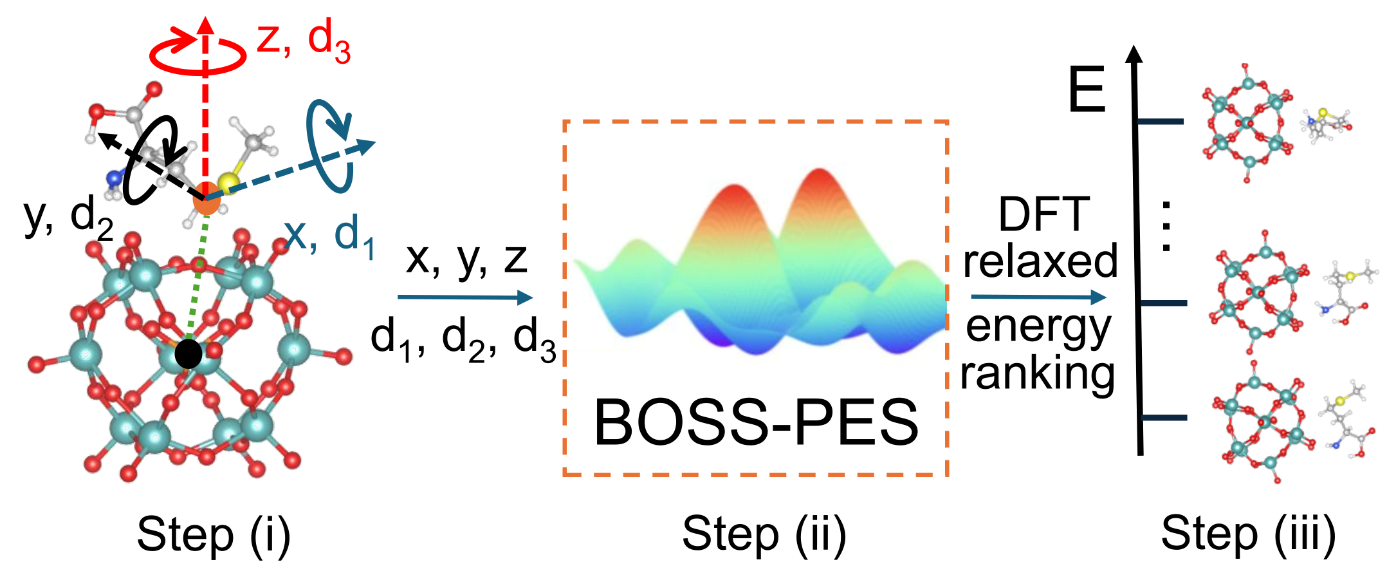


Figure S13: Overview of BOSS-based procedure for conformer search, featuring (i) system preparation, (ii) BOSS active learn PES, and (iii) DFT relax local minimum structures.

**Reference**

1. Blum, V. Ab Initio Molecular Simulations with Numeric Atom-Centered Orbitals. *Comput Phys Commun* **180**, 2175 (2009).

2. Havu, V., Blum, V., Havu, P. & Scheffler, M. Efficient O(N) integration for all-electron electronic structure calculation using numeric basis functions. *J Comput Phys* **228**, 8367 (2009).

3. Ren, X. *et al.* Resolution-of-identity approach to Hartree–Fock, hybrid density functionals, RPA, MP2 and GW with numeric atom-centered orbital basis functions. *New J Phys* **14**, 053020 (2012).

4. Perdew, J. P., Burke, K. & Ernzerhof, M. Generalized Gradient Approximation Made Simple. *Phys Rev Lett* **77**, 3865 (1996).

5. Tkatchenko, A. & Scheffler, M. Accurate Molecular Van Der Waals Interactions from Ground-State Electron Density and Free-Atom Reference Data. *Phys Rev Lett* **102**, 073005 (2009).

6. Fang, L., Guo, X., Todorović, M., Rinke, P. & Chen, X. Exploring the Conformers of an Organic Molecule on a Metal Cluster with Bayesian Optimization. *J. Chem. Inf. Model.* **63**, 745–752 (2023).
